# Brain linear measurement index in the differential diagnosis between idiopathic normal pressure hydrocephalus and progressive supranuclear palsy

**DOI:** 10.1007/s10072-025-08478-z

**Published:** 2025-10-14

**Authors:** Antonina Luca, Giulia Donzuso, Federico Contrafatto, Giovanni Mostile, Calogero Edoardo Cicero, Alessandra Nicoletti, Mario Zappia

**Affiliations:** 1 School of Medicine and Surgery, Kore University of Enna, Enna, Italy; 2 Department of Medical, Surgical and Advanced Technologies, “GF Ingrassia”, University of Catania, Via Santa Sofia 78, Catania, 95123 Italy; 3 Oasi Research Institute-IRCCS, Troina, Italy

**Keywords:** Idiopathic normal pressure hydrocephalus, Progressive supranuclear palsy,, Diagnosis, Magnetic resonance imaging

## Abstract

**Background and purpose:**

Differential diagnosis between idiopathic normal pressure hydrocephalus (iNPH) and progressive supranuclear palsy (PSP) could be challenging, due to the presence of similar clinical and neuroradiological features. Aim of this retrospective study was to differentiate iNPH from PSP by means of brain linear measurement (BLM) index.

**Materials and methods:**

All the enrolled patients underwent a 1.5-T brain-MRI and BLM index was calculated on T1-weighted images. The magnetic resonance hydrocephalic index (MRHI) was also measured. For each index, receiver operating characteristic (ROC) analyses were performed to differentiate between iNPH and PSP obtaining the area under the curve (AUC), sensitivity, specificity, and accuracy values.

**Results:**

Seventy-three patients (35 iNPH and 39 PSP) were consecutively enrolled. BLM and MRHI were significantly higher in iNPH patients than in PSP. In differentiating iNPH from PSP, the BLM index had the highest AUC (0.997) with a sensitivity of 97.1%, a specificity of 100% and an accuracy of 98.6% with an optimal cut-off value of 0.285.

**Conclusion:**

BLM may be useful in the differential diagnosis between iNPH and PSP. At an individual level, the BLM index was the most accurate measure, representing a valid, easy and reliable tool, to achieve an accurate differentiation between these two conditions.

## Introduction

Despite the distinct pathophysiological mechanisms, differentiating between idiopathic normal pressure hydrocephalus (iNPH) and progressive supranuclear palsy (PSP) may be a challenge because they share several features including parkinsonism, gait disorder, postural instability, urinary incontinence and progressive cognitive decline to overt dementia [1]. Moreover, although PSP is characterized by the peculiar vertical ocular motor slowing or palsy, in the early stages of PSP, particularly in the non-Richardson’s phenotypes, this pathognomonic sign could be missing, leading the differential diagnosis even more difficult [1].

Beside the aforementioned symptoms, previous studies have reported an overlap in neuroradiological parameters between iNPH and PSP. In particular, the “hummingbird sign”, qualitative imaging sign based on midbrain morphology on mid-sagittal plane classically described in PSP [2], has been also reported in iNPH [3, 4]. On the other hand, the Evans’ index (EI) > 0.3, linear measurement of ventriculomegaly, as well as the callosal angle (CA) and the disproportionately enlarged subarachnoid space hydrocephalus (DESH), advocated as a diagnostic neuroimaging sign of iNPH [5], may not discriminate with very good accuracy PSP from iNPH [6]. Alongside, previous studies evaluated the potential role of linear measurement, such as the temporal ratio (TR) and the parieto-occipital ratio (POR) in iNPH diagnosis [7, 8].

Due to these neuroradiological overlaps, the interest in identifying new additional MRI indexes to differentiate iNPH from PSP has been growing, introducing the Magnetic Resonance Hydrocephalic Index-MRHI [9], resembling the POR index [7, 8], the interpeduncular angle (IA), the automated ventricular volumetry (AVV) and the cortical thickness [10–13]. However, although these new indexes revealed a potential role in the differential diagnosis between iNPH and PSP, their application may be not always easy (AVV, cortical thickness and IA), sometimes requiring expertise and dedicated software, not always available in the clinical setting.

We have recently developed a new index, named Brain Linear Measurement (BLM) index, demonstrating its usefulness to reach an accurate differentiation between iNPH and Alzheimer’s disease [14]. Moreover, considering that some neurodegenerative diseases may be presented at the beginning as iNPH [15], it could be useful also to explore the potential usefulness of the BLM index in differentiating iNPH from PSP.

## Methods

This cross-sectional retrospective study was performed according to the Strengthening the Reporting of Observational Studies in Epidemiology (STROBE) Statement: guidelines for reporting observational studies.

Patients affected by probable iNPH [5] and probable PSP [1] referring to the Neurology Unit of the University Hospital “Policlinico-San Marco” of Catania from the 1 st of January 2020 to the 1 st of September 2023 were retrospectively enrolled and for each patient clinical, neuropsychological and MRI assessment were performed during the same week. The study received the approval from the local EC and all participant provided written informed consent (EC number: 72/2021/PO).

### Clinical assessment

Demographic and clinical data were collected from patient’s medical records. Patients were evaluated by movement disorders specialists with a standard neurological examination and the UPDRS–Motor Examination (UPDRS-ME). The iNPH Grading Scale (INPHGS) was calculated in iNPH patients.

#### Neuropsychological assessment

All the enrolled subjects underwent the following neuropsychological tests: global cognition (Mini Mental State Examination), episodic memory (Rey’s Auditory Verbal Learning Test), executive functioning (Frontal Assessment Battery, Verbal Fluency Test), attention (Stroop color-word test), Visuo-spatial functioning (Clock drawing test, copy of figures).

#### MRI protocol and indexes calculation

Brain MRI was performed with a 1.5 T unit (Signa HDxt, GE Medical Systems, Milwaukee, WI, USA). A 3D T1-weighted high-resolution spoiled gradient echo (SPGR) sequence with a 1.2-mm slice thickness and an isotropic in-plane resolution of 0.98 mm was acquired. Additionally, all patients underwent T2-weighted and FLAIR images to exclude morphological abnormalities.

#### MRI qualitative features

The presence of qualitative features suggestive of iNPH and PSP was evaluated in all enrolled subjects. The DESH pattern was considered positive when there were tight high convexity and medial subarachnoid spaces with enlarged sylvian fissure, evaluated on coronal T1-weighted images [5]. The hummingbird sign was assessed as midbrain tegmental atrophy together with relative increase in length of the interpeduncular fossa over that of the anteroposterior diameter of the midbrain tegmentum on midsagittal T1-weighted images [2].

#### MRI linear measurements

**Fig. 1:**
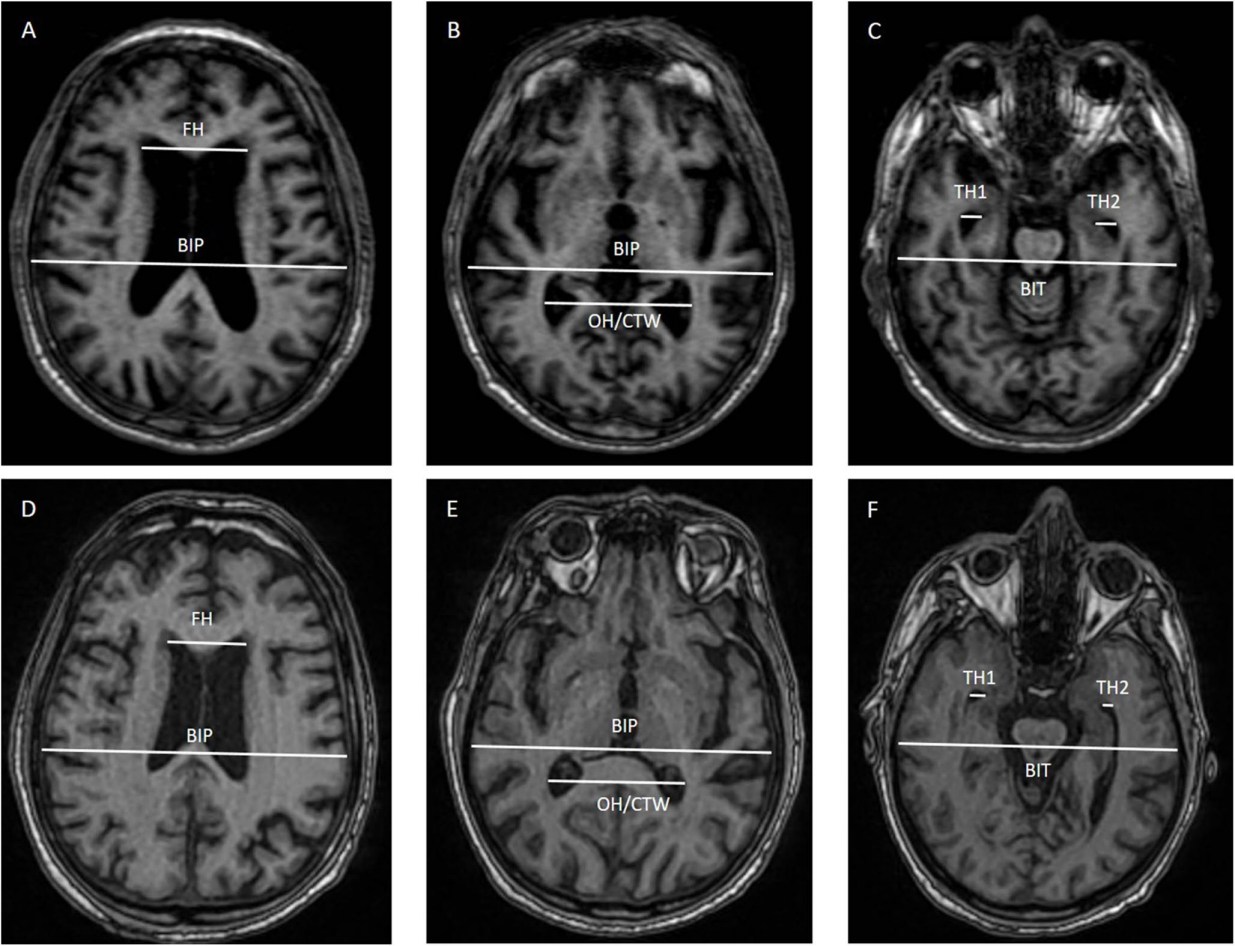
Linear measurement calculated on axial T1-weighted MRI computation of (**A**, **D**) Evan’s Index (EI), (**B**, **E**) Magnetic Resonance Hydrocephalic Index (MRHI) or Parieto-occipital Ratio (POR) and (**C**, **F**) temporal ratio (TR) to obtain Brain Linear Measurement (BLM) index for iNPH (figures above) and PSP patients (figures below). Abbreviations: FH, frontal horns; BIP: bi-parietal inner table; OH: occipital horns; CTW: collateral trigones width; TR: temporal horn; BIT: bi-temporal inner table

The BLM index was calculated in all the enrolled subjects as a cumulative index considering the linear ventricular measurements and the bi-parietal and bi-temporal diameters, as detailed elsewhere [12]. Briefly, we considered the ratio of the sum of the width of frontal horns (FH), the width of the occipital horns (OH) at the atrium and the width of the temporal horns (TH) at the level of maximal convexity of the hippocampus and the sum of the bi-parietal (BIP) and bi-temporal (BIT) diameters at same level of FH, OH and TH obtaining the following formula: (FH + OH + TH)/(BIP-FH + BIP-OH + BIT). The MRHI was also calculated considering the collateral trigones width (CTW)/inner skull diameter ratio. A MRHI > 0.579 was considered pathological [9] (Fig. 1B, E).

### Statistical analysis

Data were analyzed using STATA 18.0 software packages (Stata Statistical Software: Release 16. College Station, TX: StataCorp LLC) and Epitools Epidemiological Calculators (http://epitools.ausvet.com.au). Quantitative variables (i.e., age, disease duration, education, clinical scale scores, neuropsychological performances, MRI linear measurement) were expressed as mean and standard deviation. Qualitative variables (i.e., sex) were expressed as number and percentage. The Shapiro–Wilk normality test was performed. Differences between means were evaluated with the unpaired t-test in the case of normal distribution and the Mann–Whitney U test for not-normal distribution. The differences between proportions were evaluated by the chi-squared test. Significance was set at p value < 0.05. Additionally, a multivariate analysis, logistic regression, adjusted for sex and age was then performed to evaluate association between imaging indexes and neurological conditions (i.e., iNPH and PSP).

For the BLM index, EI and MRHI, a receiver operating characteristic (ROC) curve was developed, obtaining the area under the curve (AUC) and values of sensitivity, specificity and accuracy, and the optimal cut-off level was calculated using Youden’s method.

A further statistical analysis considering PSP phenotypes (PSP-RS versus PSP-P) was carried out.

## Results

Thirty-five patients with probable iNPH [5] and 39 patients with PSP [24 (61.5%) PSP with Richardson’s Syndrome (PSP-RS); 15 (38.5%) PSP with predominant parkinsonism (PSP-P)] [1] were enrolled. Patients with iNPH were men in the large majority (71.45 versus 38.5%) and were older than those with PSP. No statistically significant differences in terms of disease duration and global cognitive abilities explored with the MMSE were recorded comparing the two groups (Table 1). At the neuropsychological assessment, patients with iNPH presented significantly lower episodic memory performances (RAVLT, delayed recall) than patients with PSP. No other significant differences were found comparing the two groups in the other cognitive domains.

**Table 1:** Demographic, clinical and neuroradiological characteristics of the sample

	iNPH (*n*.35)	PSP (*n*.39)	*p*-value
Sex, men (*n*, %)	25 (71.4)	15 (38.5)	**0.004** ^**§**^
Age, years	74.5 ± 7.1	69.4 ± 7.7	**0.004** ^**§**^
Disease duration, years	4.5 ± 3.4	3.6 ± 2.5	0.300°
UPDRS-ME score	30.9 ± 12.7	38.1 ± 12.6	0.072^§^
INPHGS score	5.5 ± 2.1	/	/
Neuropsychological battery			
MMSE score	23.7 ± 4.1	24.3 ± 5.3	0.266°
FAB score	11.5 ± 3.7	11.5 ± 4.2	0.965^§^
HAMD score	6.1 ± 3.9	6.9 ± 5.6	0.874°
RAVLT, immediate recall score	29.7 ± 6.8	31.3 ± 9.6	0.407^§^
RAVLT, delayed recall score	4.7 ± 2.5	6.9 ± 3.4	**0.003°**
Stroop test, seconds	42.4 ± 29.0	42.0 ± 24.9	0.625°
Stroop test, errors	2.0 ± 3.0	2.1 ± 1.9	0.314°
Phonemic verbal fluency F-A-S	20.3 ± 9.3	16.8 ± 7.7	0.094^§^
Constructive apraxia, yes (n.%)	20 (57.1)	15 (38.5)	0.108^§^
MRI linear measurement			
EI	0.341 ± 0.05	0.233 ± 0.02	**< 0.001°**
MRHI	0.533 ± 0.050	0.419 ± 0.038	**< 0.001** ^**§**^
BLM	0.332 ± 0.04	0.234 ± 0.02	**< 0.001°**

Data are expressed as mean and standard deviation or number and percentage

*PSP* progressive supranuclear palsy, *iNPH* idiopathic normal pressure hydrocephalus, *MMSE: UPDRS-ME* Unified Parkinson’s Disease Rating Scale-Motor Examination, *iNPHGS* Idiopathic Normal Pressure Hydrocephalus Grading Scale; Mini Mental State Examination, *FAB* Frontal Assessment Battery, *HAM-D* Hamilton Depression Rating Scale, *RAVLT* Rey Auditory Verbal Learning Test, *EI* Evan’s Index, *BLM* brain linear measurement, *MRHI* magnetic resonance parkinsonism index

*bold values: *p*-value < 0.05. §: t-test; °: Mann-Whitney U-test

At the MRI qualitative assessment, the presence of the hummingbird sign was recorded in 16 (45.7%) iNPH and 24 (61.5%) PSP patients. Moreover, the DESH was evident in all the iNPH patients and no PSP patients (Table 1).

**Fig. 2:**
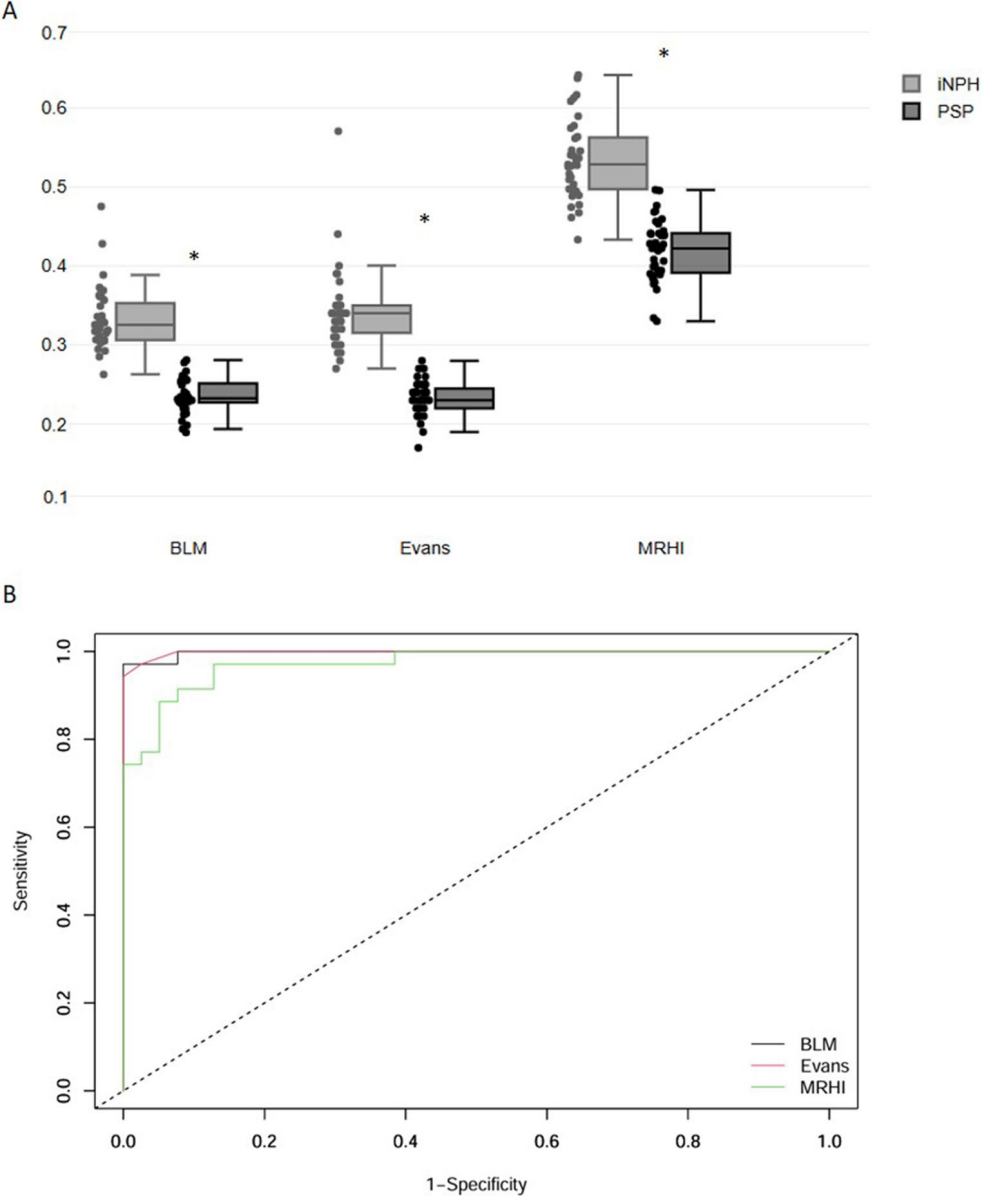
MRI Linear measurements and ROC analysis 1**A**, Box and scatter plot of BLM, Evans index and MRHI values for iNPH and PSP. 1**B**, ROC analysis showed: BLM AUC 0.998; Evans AUC 0.998; MRHI AUC 0.972.*p < 0.001. Abbreviations: BLM: Brain Linear measurement; MRHI: Magnetic Resonance Hydrocephalic Index

At the MRI linear measurement, the BLM index was significantly higher in iNPH than in PSP patients (Table 1; Fig. 2A) and an optimal cut-off value of 0.285 differentiated iNPH and PSP. Thirty-four (97.1%) iNPH and no patients with PSP had pathological BLM. The AUC was 0.998; specificity was 100%, sensitivity 97.1% and accuracy 98.6% (Fig. 2B).

EI measurement showed a significant differences between the two groups (*p* < 0.001) (Table 1; Fig. 2A). With the optimal cut-off value of 0.280, the AUC was 0.998; specificity was 97.4%, sensitivity 97.1%, and accuracy was 90.5% (Fig. 2B).

MRHI data distribution showed a slight overlap between the groups (Fig. 2A). The ROC curve analysis showed an optimal cut-off value of 0.461. Applying this cut-off, 33 (94.3%) iNPH and 5 (12.1%) PSP had a pathological MRHI. The AUC was 0.972; specificity was 87.2%, sensitivity 97.1%, and accuracy was 90.5% (Fig. 2B).

Additionally, logistic regression analysis adjusted for age and sex showed significant association between iNPH condition and MRHI (*p* = 0.005) and BLM (*p* < 0.001), while Evans showed a borderline significance (*p* = 0.063).

Considering the PSP phenotypes, the BLM was significantly higher in iNPH patients (0.332 ± 0.04) than both PSP-RS (0.230 ± 0.021; p-value < 0.001) and PSP-P (0.240 ± 0.019; p-value < 0.001).

## Discussion

In the present study, the BLM index presented a very high diagnostic accuracy, supporting its usefulness in the differential diagnosis work-up of iNPH and PSP.

Differentiating between iNPH and PSP is a fundamental step not only to prevent misdiagnosis but also to avoid useless neurosurgical procedures.

Yet, although it is well known that patients with iNPH generally exhibit ventricular enlargement, whereas PSP patients typically do not, a previous case series study found that patients who had been diagnosed with iNPH during their lifetime were reclassified as PSP following post-mortem re-examination [16]. Indeed, iNPH is still frequently misdiagnosed, often representing an early manifestation of other neurodegenerative pathologies, which become more overt over time [15]. Considering the limited knowledge on iNPH pathophysiological mechanisms, the identification of further specific neuroradiological signatures differentiating this condition from other neurodegenerative disorders should be achieved.

Indeed, as confirmed in the present study, the MRI qualitative assessment may be equivocal in these conditions. Hence, according to literature data [4, 9, 13], in this study the hummingbird sign was recorded in more than 45% of iNPH and more than 60% of PSP patients, thus supporting its limit in differentiating among these disorders. The overlap between iNPH and PSP concerns also other neuroradiological findings constantly associated with iNPH. In particular, Ohara et al. [11] reported that some patients affected by PSP presented a DESH pattern, a small CA and periventricular white matter hyperintensities similar to those observed in iNPH patients. In our study, the presence of a DESH pattern in PSP patients was not observed. Actually, the presence of a DESH pattern in PSP patients is still controversial and not frequently recorded [9]. Moreover, Önder et al. [6] in a small study enrolling 19 PSP and 18 iNPH subjects, reported that neither the neuroimaging parameters associated with PSP (i.e., the Magnetic Resonance Parkinsonism Index-MRPI and the MRPI-2.0) nor those associated with iNPH (i.e., EI, CA and DESH) revealed at the ROC curve analyses a discriminative power between the two pathologies. Thus, considering the neuroimaging overlap between PSP and iNPH, some authors have proposed new neuroimaging markers potentially useful in the differential diagnosis workup.

In particular, Caligiuri et al. [17], proposed a semi-automated approach to assess, with the MRI Diffusion Tensor Imaging, the corpus callosum microstructural integrity reporting that the alteration of the principal diffusion direction orientation (V1) in the in the midsagittal portion of the splenium could be useful in the diagnostic challenge between iNPH and PSP (AUC 0.88). However, although the V1 assessment certainly represents an interesting and sophisticated approach in the evaluation of iNPH and PSP, it requires MRI scan, an off-line workstation and an expert team to interpret the results. Ugga et al. [1] reported that the MRPI and MRPI 2.0 failed to differentiate iNPH and PSP and proposed a new measure, the interpeduncular angle (IPA), as a potential measure useful in clinical settings to differentiate these disorders. However, although IPA is easy to assess, data on its accuracy, sensibility and specificity are not available. Bianco et al. [7] performed a machine learning approach based on cortical thickness reporting a more severe and widespread cortical involvement (frontal lobe, temporal lobe, cingulate cortex, superior parietal gyrus) in iNPH than in PSP (AUC 0.95). However, the machine learning approach is certainly a complex and time-consuming procedure not suitable for clinical routine. Similarly, very recently, Georgiopoulos et al. [18] demonstrated the presence of an overlap in midbrain morphology in PSP and iNPH questioning the accuracy of MRPI in distinguishing these conditions.

Finally, Quattrone et al. [9] supported the usefulness of the automated ventricular volumetry in the differential diagnosis between iNPH and PSP and proposed the MRHI as a very accurate measure in differentiating these conditions. Considering that the MRHI, as well as the BLM Index, can be calculated not only on MRI but also on a computed tomography (CT) scan, in the present study the accuracies of both linear measurements were compared.

Interestingly, in our cohort, applying the cut-off identified by Quattrone et al. [9], the MRHI differentiated iNPH and PSP with a low accuracy (60.8%). Thus, after performing an optimal cut-off value identification in our sample, a higher accuracy of the MRHI was found (90.5%). This difference may be related to the limits of the ROC analysis whose results on a given sample of patients could not be suitable to evaluate the accuracy of the index in different cohorts in a real-word setting [19]. Although both BLM and MRHI had good accuracy, the higher diagnostic performance of BLM could be related to its intrinsic nature evaluating the whole ventricular system rather than only the dilatation of the collateral trigones, considered in the MRHI computation.

Some limits should be mentioned when interpreting our data. Due to the design of the study (hospital based and retrospective), a bias related to the selection of more advanced cases may not be ruled out. Moreover, although statistical analysis was adjusted for age and sex and the strength of the BLM index in accuracy and discrimination was confirmed, the absence of age and sex matched analysis remains a source of potential bias. Indeed, it has been demonstrated that ventricular enlargement also in normal people was related to age and sex [8], According to these data, ventricular system enlarges progressively during aging and also influenced by sex, with an abnormal consecutive geometric dilatation involving frontal horns first, followed by the most common occurrence, the enlargement of the parieto-occipital horns, and then the least frequent development, the enlargement of the temporal horns [8]. However, as for age and sex, our sample reflects the known epidemiological and phenotypic profile of iNPH [20] and PSP [21], thus increasing the generalizability and clinical applicability of our findings. Moreover, considering that our patients did not undergo shunt surgery, the diagnosis of iNPH remained “probable” and not definite. Similarly, PSP patients did not undergo autopsy, thus the lack of pathological confirmation of the diagnosis could determine a diagnostic selection bias. However, iNPH and PSP diagnoses were made using international diagnostic criteria [1, 5] and adopting a combination of clinical and radiological criteria.

Our BLM index measuring a cumulative ventricular dilatation, may overcome the limited measurement of other indexes (EI, POR, MRHI) considering only a few segments of ventricular structures. On this ground, the best accuracy of BLM as compared to other indexes, may be due to the cumulative nature itself including the whole ventricular systems. Moreover, the scarce correlation between EI and iNPH as demonstrated in our results, may further support the usefulness of BLM with respect to other measures.

Finally, in our study we investigated only linear measurement possible on both MRI and CT scan. We did not compare these linear measurements with other features, such as DESH or CA because these parameters could be better assessed only by MRI.

In conclusion, according to previous findings [12], our study further supports the usefulness of the BLM index as a valid and reliable tool, in the iNPH diagnostic work-up avoiding misdiagnosis. Moreover, of note, the BLM index can be calculated also on a CT scan and does not require specific post-processing imaging software. Larger studies are needed to confirm our findings and to identify the optimal cut-off level depending on the different iNPH mimics.

## Data Availability

The datasets generated during and/or analysed during the current study are available from the corresponding author on reasonable request.
