# Correction to: Brain linear measurement index in the differential diagnosis between idiopathic normal pressure hydrocephalus and progressive supranuclear palsy

**DOI:** 10.1007/s10072-026-08807-w

**Published:** 2026-01-10

**Authors:** Antonina Luca, Giulia Donzuso, Federico Contrafatto, Giovanni Mostile, Calogero Edoardo Cicero, Alessandra Nicoletti, Mario Zappia

**Affiliations:** 1 School of Medicine and Surgery, Kore University of Enna, Enna, Italy; 2 Department of Medical, Surgical and Advanced Technologies, “GF Ingrassia”, University of Catania, Via Santa Sofia 78, 95123 Catania, Italy; 3 Oasi Research Institute-IRCCS, Troina, Italy

**Correction to: Neurological Sciences (2025) 46:6965–6971**

10.1007/s10072-025-08478-z

In the original version of the manuscript, the authors' given and family names were swapped for all authors except the corresponding author. The correct author names are listed as follows:

Antonina Luca, Giulia Donzuso, Giovanni Mostile, Calogero Edoardo Cicero, Alessandra Nicoletti

The original article has been corrected.

